# High-quality draft genome sequences of seven *Ralstonia* spp. isolated from temperate forest soils

**DOI:** 10.1128/mra.00016-25

**Published:** 2025-04-14

**Authors:** A. Li Han Chan, Mallory J. Choudoir, Achala Narayanan, Damayanti Rodriguez-Ramos, Grace Pold, Andrew F. Billings, Kristen M. DeAngelis

**Affiliations:** 1 Department of Microbiology, University of Massachusetts Amherst 14707, Amherst, Massachusetts, USA; 2 Department of Plant and Microbial Biology, North Carolina State University 6798 , Raleigh, North Carolina, USA; 3 Department of Plant and Microbial Biology, University of Minnesota, St. Paul, Minnesota, USA; 4 Department of Bacteriology, University of Wisconsin Madison 14748, Madison, Wisconsin, USA; 5 Department of Soil and Environment, Swedish University of Agricultural Sciences 8095, Uppsala, Sweden; DOE Joint Genome Institute, Berkeley, California, USA

**Keywords:** *Ralstonia*, forest soils

## Abstract

We report seven highquality draft genomes of *Ralstonia* spp. isolated from the Harvard Forest Long-Term Warming Experimental plots: four *de novo* hybrid assemblies and three *de novo* long-read assemblies. The genomes have a minimum estimated completeness of 92.6% and an average GC content of 63.45%.

## ANNOUNCEMENT

*Ralstonia* are free-living soil bacteria known for pathogenicity (1) and terrestrial carbon cycling (2). To better understand *Ralstonia* and how they respond to climate stress, we sequenced and annotated seven *Ralstonia* isolated from experimentally heated and control soils in Prospect Hill at the Harvard Forest Long-Term Warming Experiment in Petersham, MA, USA (42.54°N, 72.18°W) (3). Soils were collected in April and June 2014 with 1/2-inch tubular soil corers to a depth of 10 cm, with the organic and mineral horizons split by eye (4). The respective isolation media and atmosphere are described in Table 1. For whole-genome sequencing, isolates were streaked from glycerol stocks onto 10% tryptic soy agar or Reasoner’s 2 agar (pH 6). Single colonies were picked to grow in the same liquid media as the agar on a shaking incubator at 30°C until the stationary phase. Genomic DNA extraction was performed on the pellets using the methods listed in Table 1. An additional RNase A treatment was performed for GP95 according to reference 5. We validated the average DNA fragment size to be 30–50 kb on 0.5% agarose gel with Quick-Load 1 kb Extend DNA ladder (New England Biolabs, Ipswich, MA, USA). For long-read sequencing using Oxford Nanopore Technologies (Oxford, UK), we followed the ligation sequencing library protocol compatible with the consumables listed in Table 1. No size selection or shearing was performed prior to library preparation. Six to seven isolates were multiplexed for a single sequencing run for 48–72 hours, with barcoded allocation turned on in the MinKNOW interface (Oxford Nanopore Technologies), except GP95, which was sequenced on a single flow cell. Fast5 or Pod5 files were base called using the high accuracy model with base callers listed in Table 1. The reads were subsampled to a minimum of 40× target coverage and controlled for quality and length using “–min_length 1000 –min_mean_q 85” on Filtlong version 0.2.1 (6). We used Flye version 2.9.1 (7) to generate the *de novo* assembly, Racon version 1.4.3 (8) and Minimap2 version 2.24 (9) to generate the consensus sequence, and Medaka version 1.7.2 (Oxford Nanopore Technologies) to generate the final polished genome draft sequence.

**TABLE 1:** Provenance with relevant citations and genome assembly statistics of each *Ralstonia* spp*.* isolate

*Ralstonia* spp*.* isolate name	AB22-23	AB28-3	AB36-13C	GP103	GP71	GP101	GP95
Soil horizon	Mineral	Mineral	Mineral	Organic	Organic	Organic	Organic
Warming treatment	Control	Warm	Warm	Warm	Control	Warm	Control
Isolation media	1% nutrient broth (10)	1% nutrient broth (10)	1% nutrient broth (10)	VL55 plant polymer (11)	VL55 plant polymer (11)	VL55 plant polymer (11)	VL55 plant polymer (11)
Isolation atmosphere	5% hydrogen, 5% CO_2_, and 90% nitrogen	Aerobic	Aerobic-COY fluctuating (4)	Aerobic	Aerobic	Aerobic	Aerobic
DNA extraction method	Qiagen DNeasy Blood & Tissue Kit (Germantown, MD, USA)	Qiagen DNeasy Blood & Tissue Kit (Germantown, MD, USA)	Qiagen DNeasy Blood & Tissue Kit (Germantown, MD, USA)	Qiagen DNeasy Blood & Tissue Kit (Germantown, MD, USA)	Qiagen DNeasy Blood & Tissue Kit (Germantown, MD, USA)	Qiagen DNeasy Blood & Tissue Kit (Germantown, MD, USA)	Modified CTAB-lysozyme extraction protocol (12, 13)
Sequencing facility	SeqCenter, Pittsburgh, PA, USA, and inhouse ONT	SeqCenter, Pittsburgh, PA, USA, and inhouse ONT	SeqCenter, Pittsburgh, PA, USA, and inhouse ONT	SeqCenter, Pittsburgh, PA, USA, and inhouse ONT	Inhouse ONT	Inhouse ONT	Inhouse ONT
Sequencing technology	Illumina NextSeq 2000 and ONT	Illumina NextSeq 2000 and ONT	Illumina NextSeq 2000 and ONT	Illumina NextSeq 2000 and ONT	ONT	ONT	ONT
ONT Flow Cell	FLO-MIN106	FLO-MIN106	FLO-MIN106	FLO-MIN106	FLO-MIN106	FLO-MIN106	FLO-MIN114
ONT Library Preparation Kit	SQK-LSK109, EXP-NBD104	SQK-LSK109, EXP-NBD104	SQK-LSK109, EXP-NBD104	SQK-LSK109, EXP-NBD104	SQK-LSK109, EXP-NBD104	SQK-LSK109, EXP-NBD104	SQK-LSK114
Base caller	Guppy version 6.5.7	Guppy version 6.5.7	Guppy version 6.5.7	Guppy version 6.5.7	Guppy version 6.5.7	Guppy version 6.5.7	Dorado version 0.7.3
BioSample	SAMN33771254	SAMN33771256	SAMN33771258	SAMN33771263	SAMN33771268	SAMN33771262	SAMN44697474
SRA accession no. (ONT)	SRX19837939	SRX19837949	SRR24035310	SRX19837956	SRX19837945	SRX19837955	SRX26711341
SRA accession no. (Illumina)	SRX26601167	SRX26601168	SRX26601169	SRX26601170	N/A^*a*^	N/A^*a*^	N/A^*a*^
WGS accession no.	JBJKIK000000000	JBJKIJ000000000	JBJKII000000000	JBJKIG000000000	JBJKIF000000000	JBJKIH000000000	JBJKIE000000000
Assembly year	2022	2022	2022	2022	2022	2021	2024
Assembly type	*de novo* hybrid	*de novo* hybrid	*de novo* hybrid	*de novo* hybrid	*de novo* long read	*de novo* long read	*de novo* long read
Reads Illumina (read pairs)	3,625,687	2,957,812	3,160,759	3,519,480	N/A	N/A	N/A
Number of raw ONT reads (bp)	149,100,000	220,000,000	246,700,000	220,000,000	2,100,000,000	5,645,342,330	6,011,438,671
Sequencing *N*_50_ for ONT (bp)	12,636	17,948	10,557	24,364	43,073	16,001	4,038
Filtered reads ONT (bp)	149,072,979	488,529,099	246,663,274	554,542,900	240,016,725	216,041,805	236,625,424
Genome size (bp)	5,298,867	5,483,600	5,513,759	5,515,304	5,432,770	5,513,656	5,346,046
Coverage	184.9	151.1	160.9	179.0	44.2	39.2	44.3
Assembly *N*_50_ values (bp)	1,386,635	3,507,490	3,537,649	3,537,649	3,537,633	3,537,632	3,533,349
GC content (%)	63.57	63.61	63.58	63.58	62.63	63.58	63.63
No. of contigs	7	6	5	6	4	5	6
Contamination (%)	0.47	0.47	0.47	0.47	0.47	0.47	0
Completeness (%)	92.6	99.94	99.94	99.94	99.06	98.92	99.94
Most closely related genome according to whole genome sequence in NCBI (% average nucleotide identity)	*Ralstonia thomasii* (99.72%)	*Ralstonia thomasii* (99.73%)	*Ralstonia thomasii* (99.73%)	*Ralstonia thomasii* (99.73%)	*Ralstonia thomasii* (99.79%)	*Ralstonia thomasii* (99.72%)	*Ralstonia thomasii* (99.73%)
23S rRNA genes count	21	21	21	21	20	21	21
16S rRNA genes count	17	19	19	19	19	16	19
5S rRNA genes count	3	3	3	3	3	3	3
trRNA count	64	78	78	78	65	86	78

^*a*^ N/A, not applicable as these strains were assembled with ONT reads only.

For *de novo* hybrid assembled isolates, short read libraries were constructed using Illumina (San Diego, CA, USA) DNA Prep kit and IDT (Coralville, IA, USA) 10 bp UDI indices and sequenced on NextSeq 2000 (Illumina) to produce 2 × 151 bp reads. Demultiplexing, quality control, and adapter trimming were performed with bcl-convert version 3.9.3 (Illumina). *De novo* hybrid assemblies were generated using Unicycler version 0.5.0 (14). Default parameters were used except where otherwise noted.

Genomes were identified as *Ralstonia* with average nucleotide identity by NCBI (15). The genome sizes for these isolates range from 5,298,867 to 5,515,304 bp, with GC content ranging from 62.63% to 63.63%. Genome contamination and completeness were estimated with CheckM version 1.0.18 (16). Genomes were not circularized and were annotated with RASTtk version 1.073 (17).

## Data Availability

This project has been deposited in GenBank under the accession number PRJNA944978. The respective BioSample numbers, SRA accession numbers for Oxford Nanopore Technologies and Illumina sequencing (where applicable), and WGS accession numbers are listed in Table 1.
